# Disodium Cromoglycate Attenuates the Depressive‐Like Behaviors in Mice by Inhibiting Neuroinflammation

**DOI:** 10.1002/cns.70721

**Published:** 2026-01-20

**Authors:** Yun Xiao, Kaifan Liu, Zengqiang Yuan, Yajin Liao

**Affiliations:** ^1^ The Brain Science Center Beijing Institute of Basic Medical Sciences Beijing China; ^2^ Department of Neurology, the Second Affiliated Hospital, Hengyang Medical School University of South China Hengyang China; ^3^ NHC Key Laboratory of Neurodegenerative Diseases (University of South China) Hengyang China

**Keywords:** disodium cromoglycate, major depression, mast cell, neuroinflammation

## Abstract

**Aims:**

Emerging evidence indicates that mast cells (MCs) may play a crucial role in the pathogenesis of major depression disorder (MDD). This study aimed to investigate whether the mast cell membrane stabilizer Disodium cromoglycate (DSCG) could ameliorate depressive‐like behaviors by attenuating mast cell‐mediated neuroinflammation.

**Methods:**

Lipopolysaccharide (LPS)‐induced and chronic restraint stress (CRS)‐induced mouse models were induced in C57BL/6 mice to evaluate the therapeutic effect of the DSCG. Depressive‐like behaviors were assessed using the sucrose preference test (SPT), tail suspension test (TST), and forced swimming test (FST). Histopathological and molecular changes were examined through immunofluorescence, western blot, RT‐qPCR, and ELISA.

**Results:**

Firstly, our results indicated that the number of MCs was increased in the brain from LPS‐induced depression model mice. Secondly, both CRS and LPS‐induced depressive‐like behaviors were significantly ameliorated by DSCG. Moreover, treatment with DSCG could down‐regulate the expression of MCs‐associated genes in the brain of depression model mice. Mechanically, our results displayed that the use of DSCG significantly suppressed the activation of glial cells and the expression of pro‐inflammatory factors.

**Conclusion:**

Our study demonstrates that MCs infiltration and activation contribute to neuroinflammation in LPS‐induced depressive mice. DSCG exerts its antidepressant effects primarily by modulating MCs‐mediated neuroinflammation. These results highlight DSCG as a promising therapeutic candidate for the treatment of inflammation‐associated depression.

## Graphical abstract

Mast cells were increased in the brain of depression model mice. Administration of Disodium Cromoglycate could alleviate depressive‐like behaviors in mice of LPS and CRS models. Administration of Disodium Cromoglycate mitigated the infiltration of mast cells in brain and down‐reuglated the expression of pro‐inflammatory factors in the brain of depression model mice.

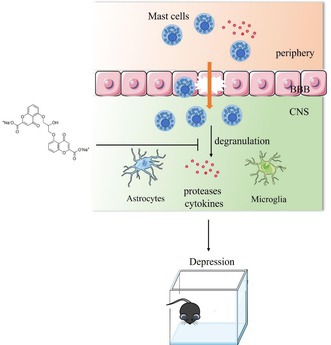

## 1 Introduction

Major depression represents a profoundly disabling public health concern, characterized by persistent feelings of sadness and hopelessness, and has affected a large global population [1]. Currently, it affects over 264 million individuals across all age groups [2], with women exhibiting approximately twice the incidence rate of men [3]. Major depression is attributable not only to elevated suicide risk but also to a marked increase in the incidence of comorbid conditions such as cardiovascular diseases, stroke, autoimmune disorders, diabetes, and cancer [4, 5, 6]. Despite decades of research, effective antidepressant treatments remain limited, and many patients fail to achieve full remission.

Systemic immune activation is found to be associated with major depression, evidenced by abnormalities in inflammatory markers, alterations in immune cell populations, and dysregulated antibody titers [7, 8]. Patients with major depressive disorder (MDD) display elevated serum levels of IL‐1β and IL‐18, accompanied by increased expression of the NLRP3 inflammasome and caspase‐1 [9]. Mast cells (MCs) are multifunctional immune cells that release a wide array of pro‐inflammatory mediators, including histamine, tumor necrosis factor‐α (TNF‐α), and β‐tryptase [10, 11]. The number of MCs in the brain is found to be increased under psychological stress [12, 13]. Under environmental stress, both the number and degranulation status of brain MCs undergo significant changes, thereby exacerbating neuroinflammation implicated in depression pathogenesis. Mastocytosis is a disease that patients display increased MCs, of which 60% of patients exhibit depressive symptoms [14]. Intriguingly, inhibition of the proliferation and activation of MCs with masitinib could also alleviate the depressive symptoms in mastocytosis patients [15, 16]. In addition, MCs not only respond rapidly to inflammatory stimuli but also communicate with neighboring cells via degranulation, thereby amplifying microglial activation [17]. For instance, suppressing MCs' degranulation by the MCs stabilizer disodium cromoglycate (DSCG) has been shown to attenuate the activation of microglia [17]. However, the role and mechanism of MCs in the development of MDD remain unknown.

Pronounced microglial activation has been observed in the prefrontal cortex (PFC) and anterior cingulate cortex (ACC) during severe depressive episodes, with the degree of activation levels in the ACC positively correlating with depressive symptom severity [18]. Mechanically, the NLRP3 inflammasome, a multiprotein complex comprising NLRP3, ASC, and pro‐caspase‐1, plays a pivotal role in stress‐induced microglial activation [19]. Under chronic stress, NLRP3 activation triggers caspase‐1‐dependent cleavage of pro‐IL‐1β and pro‐IL‐18 in microglia, exacerbating neuroinflammatory responses linked to MDD pathogenesis [20, 21]. Consistently, LPS‐induced depressive‐like behavior in mice is accompanied by upregulation of NLRP3, ASC, and caspase‐1 mRNA [22], whereas pharmacological inhibition of NLRP3 reverses these behavioral phenotypes [23, 24]. Moreover, established antidepressants, including minocycline and fluoxetine, exert part of their therapeutic efficacy through suppressing NLRP3 activation [25, 26, 27, 28, 29]. Notably, several clinical antidepressants exert their therapeutic effects by modulating microglial activity and attenuating neuroinflammation [30, 31].

In the current study, we demonstrate that the amounts of MCs were increased in the brain from depression model mice, and administration of DSCG could ameliorate depressive‐like behaviors in both LPS‐induced and CRS mouse models. Notably, DSCG treatment significantly down‐regulated the expression of mast cell marker genes in the brains from depressed mice, therebydecreasing the number of microglia and astrocytes. Mechanically, we reveal that DSCG modulates depressive‐like behaviors through down‐regulating the inflammatory response in the brain. In conclusion, our study proved that inhibition of MCs with DSCG could attenuate the depressive‐like behaviors by suppressing neuroinflammation, suggesting DSCG is a new candidate for the treatment of depression.

## 2 Material and Methods

### 2.1 Mice

8‐week‐old C57BL6/N mice (5 per cage) were raised in the Animal Care Facility at the Institute of Basic Medical Sciences. They all live in an ambient temperature of 22°C and a light exposure period of 12 h. During the experiment, mice could freely obtain food and water.

### 2.2 Experimental Design for Drug Treatment

#### 2.2.1 LPS‐Induced Depressive Mice Model

The mice were divided into four groups: ctrl, LPS, LPS + DSCG (1 mg/kg), LPS + DSCG (2 mg/kg). The experimental design and drug treatment plan are shown in Figure 2A. Firstly, DSCG was intraperitoneally injected for two consecutive days. On the third and fourth days, LPS (0.5 mg/kg) was injected intraperitoneally 1 h after DSCG treatment. On the fifth day, behavioral assessments were performed 1 h following DSCG administration. Finally, at the end of the experiment, the mice were sacrificed and the brains were taken out; the cortex and hippocampus were collected for further study.

#### 2.2.2 CRS‐Induced Depressive Mice Model

CRS is based on previous reports and has undergone minor modifications [19]. The experimental design and drug treatment plan are shown in Figure 3A. In detail, the mice were divided into four groups: ctrl, CRS, CRS + DSCG (1 mg/kg), CRS + DSCG (2 mg/kg). For the CRS procedure, mice were individually placed in a 50 mL conical tube for 4 h every day. The conical tube contained 0.5 cm Wells. One hour post CRS treatment, DSCG was administered intraperitoneally. This protocol was continuously applied for 14 days.

### 2.3 Open Field Test (OFT)

First, let the mice adapt to the test environment for 1 h, then place the mice in the device (50 × 50 × 20 cm) and move freely for 5 min. The time spent in the central zone and the total distance traveled were quantified using ANY‐maze software.

### 2.4 Sucrose Preference Test (SPT)

The sucrose preference test was conducted to assess anhedonia in mice. The double‐bottle free selection method is adopted. On the first day, both bottles were filled with water. On the second day, they were filled with 2% sucrose. Following 12 h of water deprivation, the test commenced. Each mouse can freely choose a bottle containing sucrose and water. The positions of the bottles were alternated every 2 h to avoid side preference. Twelve hours later, the intake of sucrose and water was recorded and calculated, and the sucrose preference rate was calculated. The formula is: (sucrose consumption/sucrose consumption + water consumption) × 100%.

### 2.5 Tail Suspension Test (TST)

By attaching medical tape 1 cm away from the tip of the tail, the mice were inverted at a position about 50 cm above the ground. The test was conducted for 6 min, and the duration of immobility during the final 4 min was recorded. Behavioral recording and analysis of TST were performed using Smart 3.0 software.

### 2.6 Forced Swimming Test (FST)

The mice were placed in a transparent glass cylinder with a diameter of 25 cm, and the water temperature inside the cylinder was maintained at 21°C to 23°C. The water level reached 25 cm above the bottom. The entire test lasted for 6 min, and the immobile time of the mice was recorded in the last 4 min. Videos were recorded and analyzed using the Smart 3.0 software.

### 2.7 Quantitative Real‐Time Polymerase Chain Reaction

Total RNA was isolated from tissues using 1 mL Trizol reagent (15,596,018, Invitrogen, USA). The first‐strand cDNA was synthesized from 1 μg of total RNA using the One‐step first‐strand cDNA Synthesis Kit (AE311‐03; Transgen, China). A 10‐fold dilution of the cDNA by ddH_2_O was performed before the assay. Gene expression was detected by qPCR based on SYBR‐green master mix. The primer sequences used are shown in Table S1. The mRNA expression level was normalized to β ‐actin.

### 2.8 Enzyme‐Linked Immunosorbent Assay

For brain tissue samples, the brain was lysed with RIPA lysis buffer containing a mixture of protease and phosphatase inhibitors. The samples were assayed undiluted for IL‐1β, and at a 3‐fold dilution for TNF‐α and IL‐6, respectively. Then, an enzyme‐linked immunosorbent assay (ELISA) kit was used to detect the concentrations of IL‐1β (abs552801; Absin), TNF‐α (430,904; Biolegend), and IL‐6 (431,304; Biolegend) in accordance with the manufacturer's instructions.

### 2.9 Immunohistochemistry

The brains were fixed in 4% fixator (P1110; Solarbio), dehydrated in a graded sucrose series, and embedded in the optimal cutting temperature compound (OCT) (4583; SAKURA). Coronal sections (40 μm) were cut on a freezing microtome (CM3050S; Leica) and stored at −20°C. Immunofluorescence staining was performed as previously described [32]. The slides were stained with specific primary antibodies: anti‐goat AIF‐1/Iba1 (1:200, NB100‐1028; Novus), anti‐mouse GFAP (1:500, MAB360; Millipore), anti‐rabbit ASC (1:200, F0468; Selleck), FITC anti‐mouse CD117 Antibody (1:200, 135,115; Biolegend). All fluorescent secondary antibodies are purchased from Jackson ImmunoResearch: Alexa Fluor 647 AffiniPure donkey anti‐goat IgG (1:500; 705–605‐147), Alexa Fluor 488 AffiniPure donkey anti‐mouse IgG (1:500; 715–545‐150), TRITC AffiniPure donkey anti‐rabbit IgG (1:500; 711–025‐152). The images were acquired using a confocal microscope (Nikon).

### 2.10 Western Blotting

The brain tissue was lysed with RIPA lysis buffer containing a mixture of protease and phosphatase inhibitor (HY‐K0021; MedChemExpress). The samples were centrifuged at 14,000 rpm for 15 min in 4°C, and the supernatant was collected. The protein concentration in the lysis buffer was determined by the Bicinchoninic Acid Assay (BCA) method (P0009; Beyotime) and adjusted to the same final concentration. Heat at 95°C for 15 min to denature the protein. Prepare 12% separation gel and 5% concentrated gel. After transfer, seal at room temperature with 5% skimmed milk for 2 h to reduce non‐specific binding. Then incubate overnight with the primary antibody. Subsequently, the secondary antibody labeled with horseradish peroxidase (HRP) was incubated, and the immunoreactive protein was detected by enhanced chemiluminescence (ECL) substrate. The primary antibody including: anti‐GAPDH (1:3000, GB15004‐100; Servicebio), anti‐AIF‐1/Iba1 (1:1000, NB100‐1028; Novus), anti‐GFAP (1:1000, MAB360; Millipore), anti‐β‐actin (1:5000, GB15003‐100; Servicebio), anti‐ASC (1:1000, F0468; Selleck), anti‐IL‐1β (1:1000, AF‐401‐NA; R&D systems), anti‐caspase 1 (1:1000, Ag‐20B‐0042; Adipogen). The protein band intensities were quantified using ImageJ and normalized to the corresponding loading control.

### 2.11 Statistical Analysis

All data were expressed as mean ± SEM. Data analysis was conducted using GraphPadPrism8.0 software. The significance of the difference was evaluated using the unmatched student *t*‐test. The statistical significance levels were set as: *p < 0.05, **p < 0.01, ***p < 0.001, ****p < 0.0001.

## 3 Results

### 3.1 Mast Cells Were Increased in the Brain From LPS‐Induced Depression Model Mice

**FIGURE 1:**
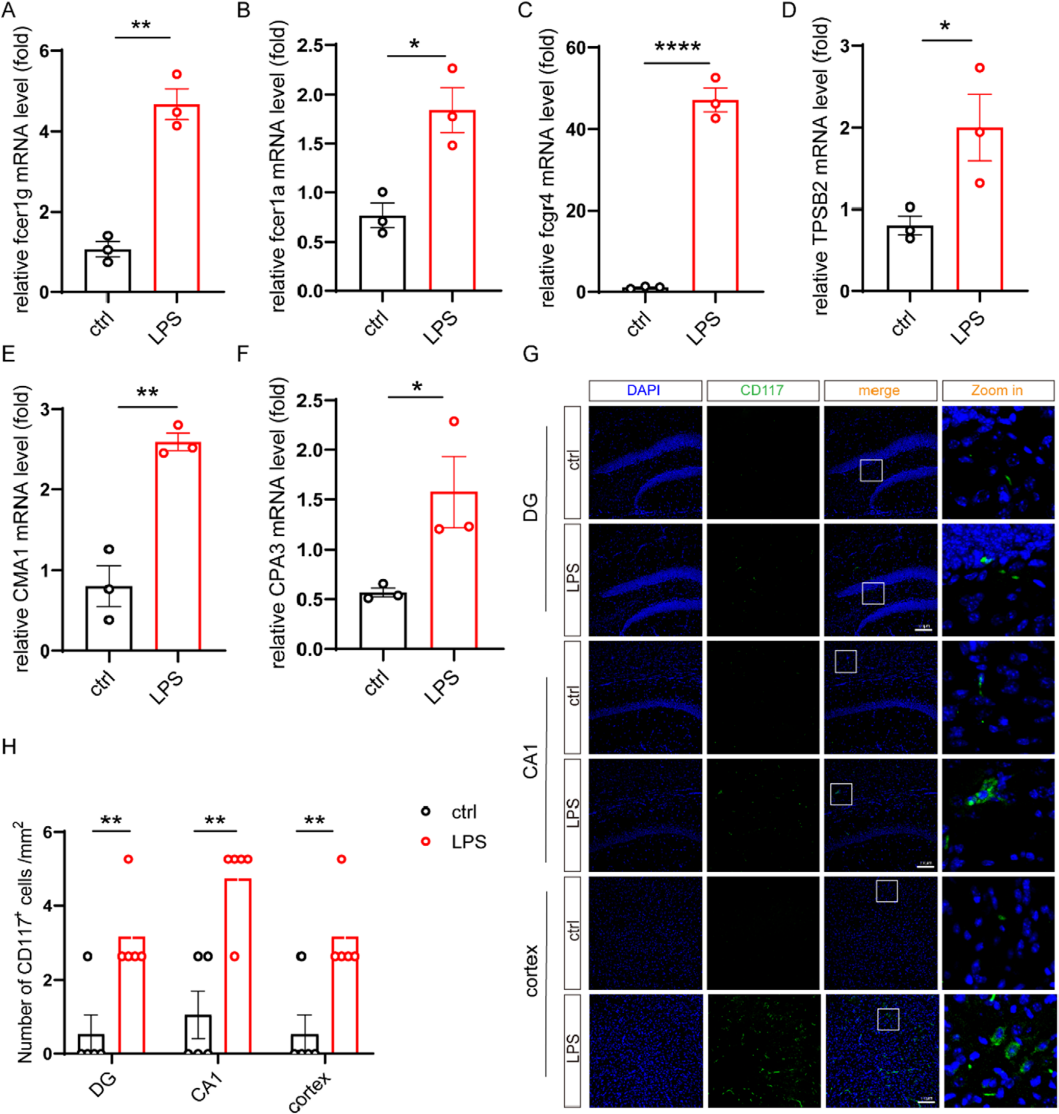
Mast cells are increased in the brain of LPS‐induced depression model mice. (A–F) The mRNA levels of Fcer1g, Fcer1α, Fcgr4, TPSB2, CMA1, and CPA3 in the cortex from ctrl (*n* = 3) and LPS‐induced mice (*n* = 3) were determined by qPCR. (G) Representative immunofluorescence staining for CD117 in the DG, CA1, and cortex. Scale bars, 100 μm. (H) Quantification of CD117^+^ cells, including ctrl (*n* = 5), LPS (*n* = 5). Data are mean ± SEM. *p < 0.05, **p < 0.01, ***p < 0.001, and ****p < 0.0001. Two‐tailed unpaired Student's *t* test.

The character of MCs is expressing high levels of the high‐affinity IgE receptor FcεRI, which is a tetrameric complex composed of one FcεRIα chain, one FcεRIβ chain, and two FcεRIγ chains [33]. To clarify whether MCs is involved in the development of major depression, we firstly investigated the number of MCs in the brain from LPS‐induced depression model mice. The results showed that the expression of fcer1g, fcer1a, and fcgr4 was significantly increased (Figure 1A–C). Consistently, the mRNA levels of mast cell‐specific proteases, such as Chymase 1 (CMA1), Carboxypeptidase A 3 (CPA3), and Tryptase Beta 2 (TPSB2) were also upregulated in the brain from depression model mice (Figure 1D–F). Moreover, the tyrosine kinase CD117 is a type III, transmembrane receptor that is specifically expressed in MCs, melanocytes, and neoplasms of these cells [34]. Immunofluorescence staining revealed the presence of a small number of CD117^+^ cells in various brain regions of mice, including dentate gyrus (DG), Cornu Ammonis 1 (CA1), and Pre‐Frontal Cortex, indicating that MCs can infiltrate the CNS in LPS‐induced depression model (Figure 1G,H). These findings collectively suggest the amounts of MCs are increased in the brain of LPS‐induced depression model mice.

### 3.2 Administration of DSCG Could Significantly Ameliorate the Depression‐Like Behaviors in LPS‐Induced Mice

**FIGURE 2:**
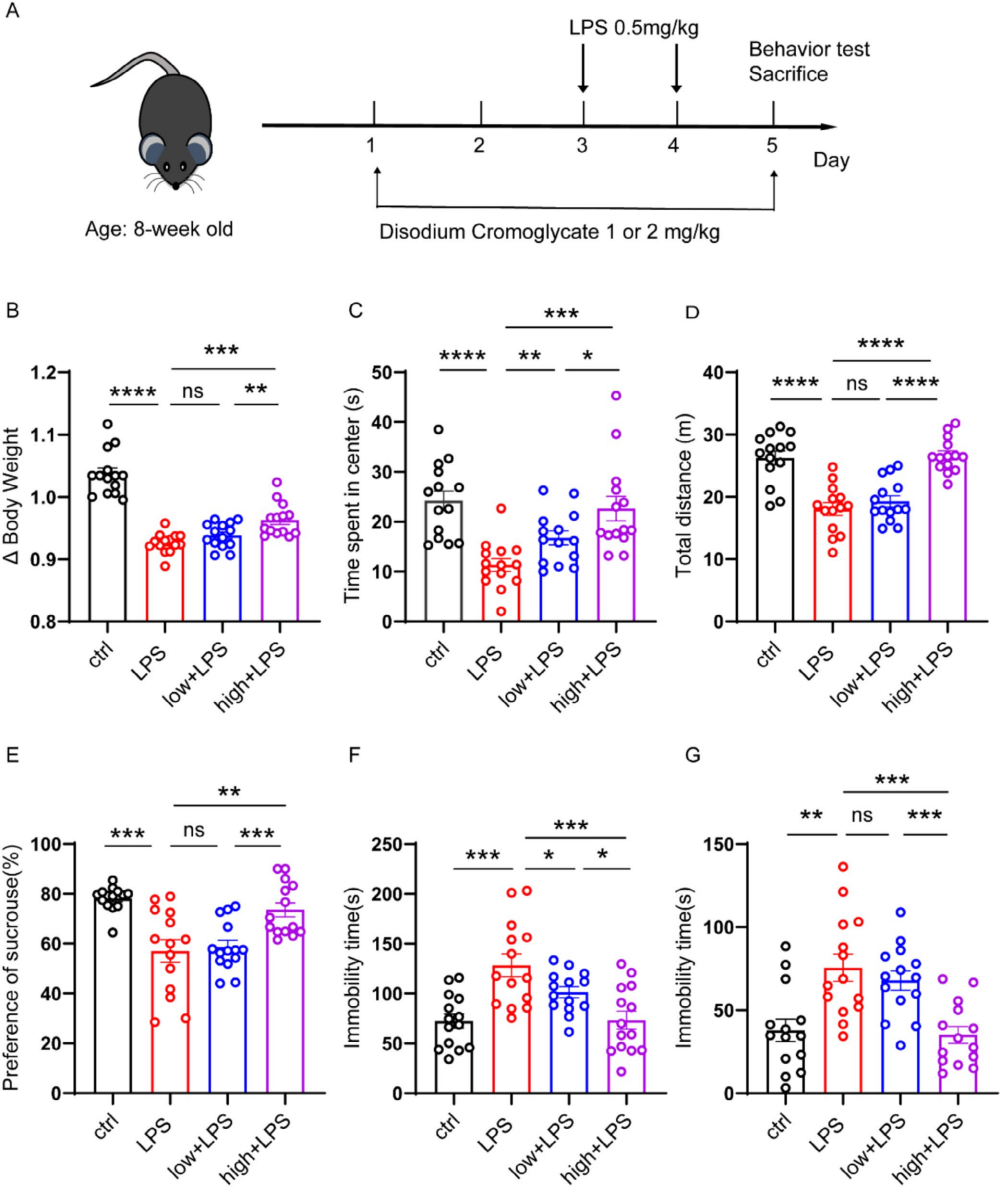
Administration of DSCG could ameliorate the depression‐like behaviors in LPS‐induced mice. (A) Drug treatment schedule. (B) Body Weight of mice in different groups: Ctrl (*n* = 14), LPS (*n* = 14), 1 mg/kg DSCG + LPS (low + LPS) (*n* = 14) and 2 mg/kg + LPS (high + LPS) (*n* = 14). (C, D) Time spent in the center and total travel distance in the OFT. (E) Sucrose preference of mice in different groups: Ctrl (*n* = 14), LPS (*n* = 14), 1 mg/kg DSCG + LPS (low + LPS) (*n* = 14) and 2 mg/kg + LPS (high + LPS) (*n* = 14). (F, G) Immobility duration in the TST and FST. Data are presented as the mean ± SEM; *p < 0.05; **p < 0.01, ***p < 0.001, and ****p < 0.0001. Two‐tailed unpaired Student's *t* test.

We then aimed to investigate whether inhibition of MCs with DSCG could attenuate the depressive behaviors in mice. The experimental design is illustrated in Figure 2A, and the open field test (OFT), sucrose preference test (SPT), tail suspension test (TST), and forced swim test (FST) were used to assess the depressive behaviors. As expected, LPS‐treated mice exhibited a significant reduction in body weight compared with controls (Figure 2B). In addition, mice injected with LPS showed a significant reduction in the total distance and the time spent in the center in the OFT (Figure 2C,D). Furthermore, LPS‐injected mice displayed a pronounced decrease in sucrose preference in the SPT, along with prolonged immobility durations in both the TST and FST (Figure 2E–G). Interestingly, DSCG treatment effectively reversed these behavioral alterations in a dose‐dependent manner (Figure 2B–G).

### 3.3 Administration of DSCG Alleviates Depression‐Like Behaviors in Mice Induced by CRS

**FIGURE 3:**
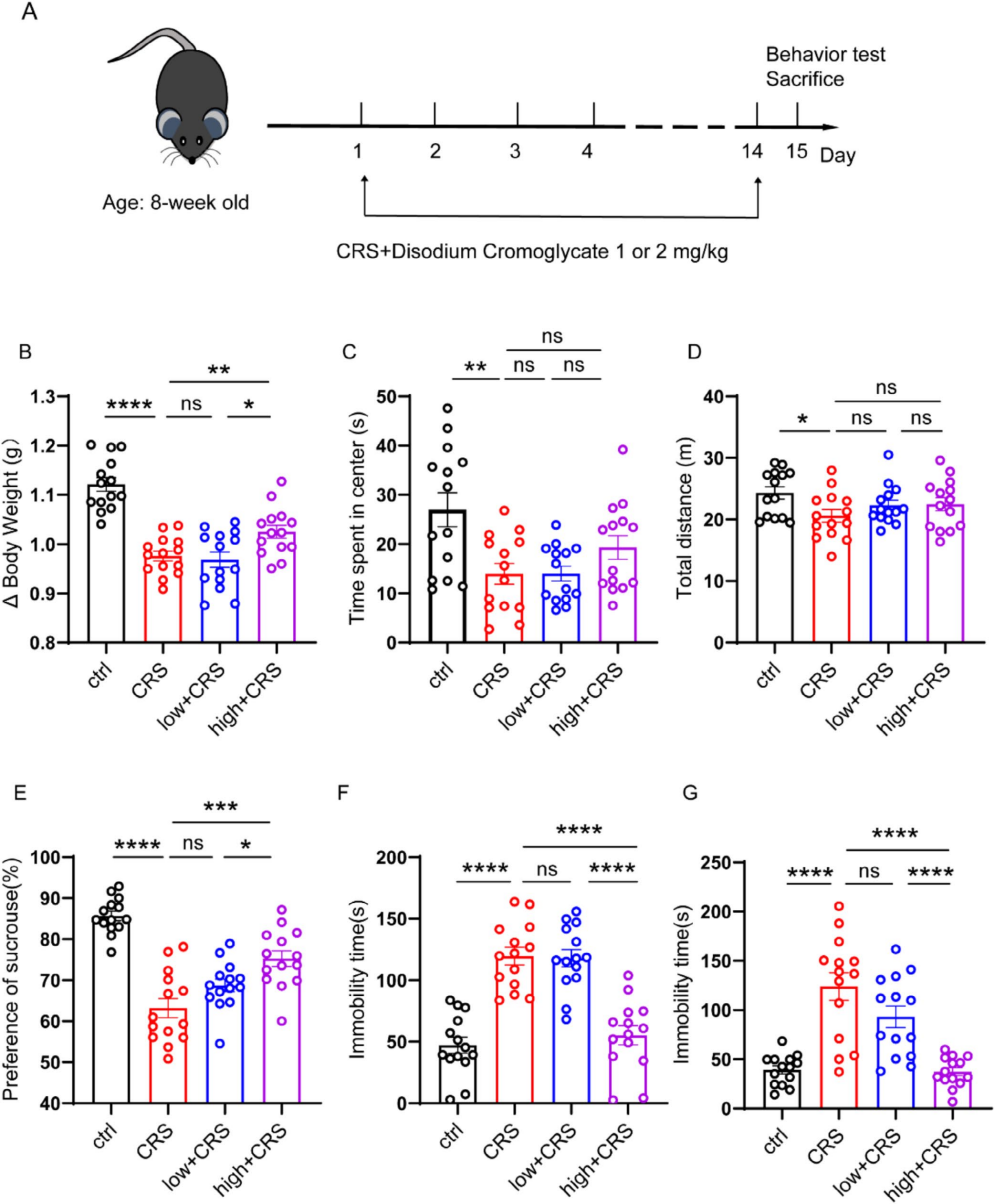
Administration of DSCG could ameliorate the depression‐like behaviors in CRS‐induced mice. (A) Drug treatment schedule. (B) Body Weight of mice in different groups: Ctrl (*n* = 14), CRS (*n* = 14), 1 mg/kg DSCG + CRS (low + CRS) (*n* = 14) and 2 mg/kg + CRS (high + CRS) (*n* = 14). (C, D) Time spent in the center and total travel distance in the OFT. (E) Sucrose preference of mice in different groups: Ctrl (*n* = 14), CRS (*n* = 14), 1 mg/kg DSCG + CRS (low + CRS) (*n* = 14) and 2 mg/kg + CRS (high + CRS) (*n* = 14). (F, G) Immobility duration in the TST and FST. Data are presented as the mean ± SEM; *p < 0.05; **p < 0.01, and ****p < 0.0001. Two‐tailed unpaired Student's *t* test.

The above results suggest that DSCG may play an anti‐depression role; then, we assessed the anti‐depression ability of DSCG in the CRS‐induced depression model, another widely adopted method for studying depression [35]. The experimental plan is shown in Figure 3A. Briefly, mice were randomly assigned to four groups. The CRS group underwent daily restraint stress, whereas control mice were housed individually for an equivalent duration. Following 4 h of restraint stress, mice were released and allowed free movement. After 1 h, they were administered different doses of DSCG (1 or 2 mg/kg). Behavioral assessments, including the SPT, OFT, TST, and FST, were conducted on day 15 (Figure 3A). CRS exposure resulted in a significant reduction in body weight (Figure 3B). Behavioral test results show that CRS‐treated mice exhibited decreased exploratory activity, as evidenced by reduced time spent in the center and diminished total distance traveled in the OFT (Figure 3C,D). Additionally, CRS‐induced pronounced anhedonia (Figure 3E) and increased despair‐like behavior (prolonged immobility in TST and FST) (Figure 3F,G). Notably, DSCG administration (2 mg/kg) significantly attenuated these effects, restoring body weight (Figure 3B), increasing sucrose consumption (Figure 3E), and reducing immobility time in both TST and FST (Figure 3F,G) in a dose‐dependent manner. Collectively, these findings indicate that DSCG exerts robust antidepressant‐like effects in the CRS‐induced depression model, further supporting its therapeutic potential for stress‐related depressive disorders.

### 3.4 Administration of DSCG Could Down‐Regulate Mast Cell‐Associated Genes in the Brain of Depression Model Mice

**FIGURE 4:**
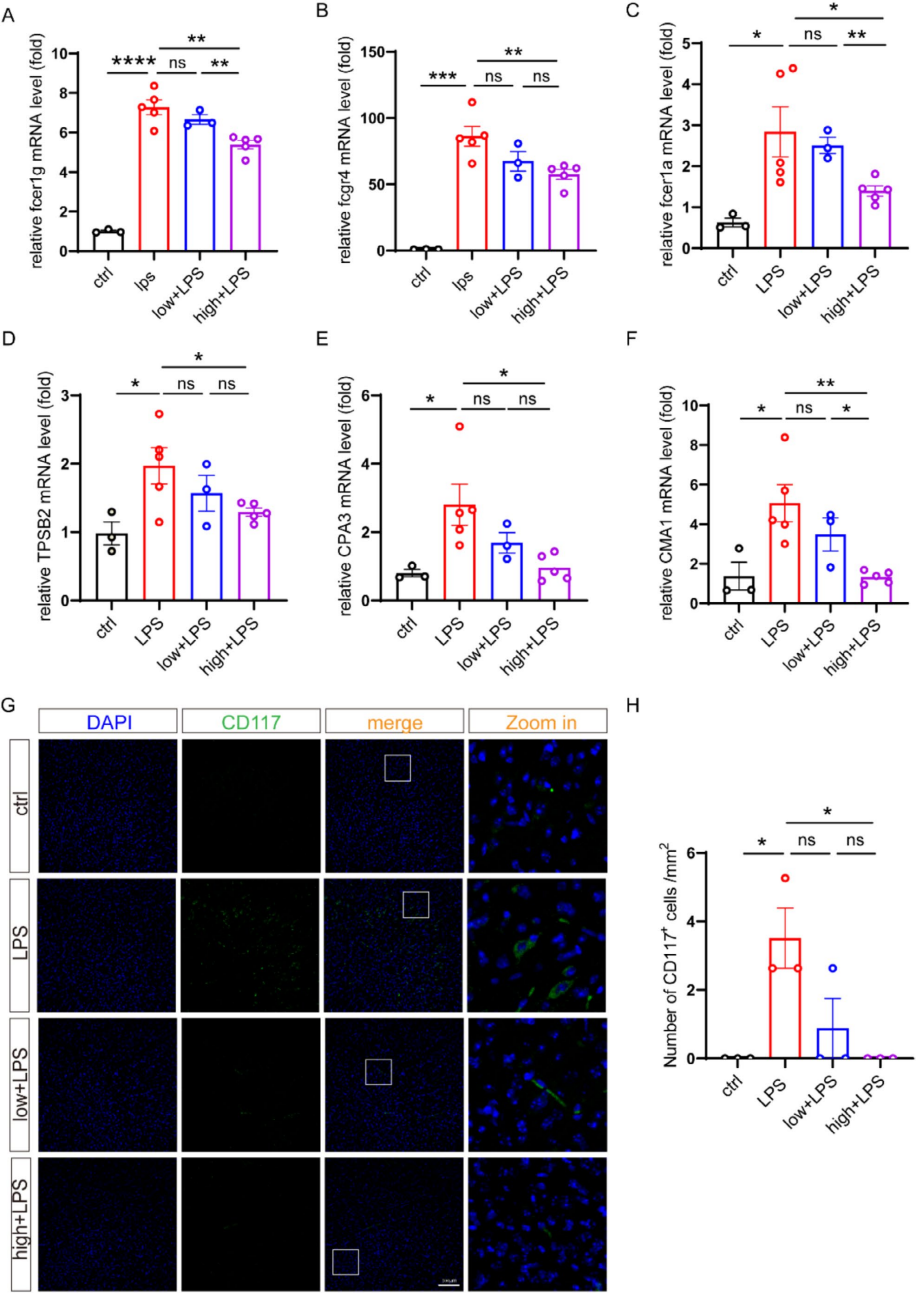
Administration of DSCG could down‐regulate mast cell‐associated genes in the brain of depression model mice. (A–F) The mRNA levels of Fcer1g, Fcer1α, fcgr4, TPSB2, CMA1 and CPA3 in the cortex from ctrl (*n* = 3), LPS (*n* = 5), 1 mg/kg DSCG + LPS (low + LPS) (*n* = 3) and 2 mg/kg + LPS (high + LPS) (*n* = 5) mice were determined by qPCR. (G) Representative immunofluorescence staining for CD117 in the cortex. Scale bars, 100 μm. (H) Quantification of CD117^+^ cells, including ctrl (*n* = 3), LPS (*n* = 3), low + LPS (*n* = 3), high + LPS (*n* = 3). Data are mean ± SEM. *p < 0.05, **p < 0.01, ***p < 0.001 and ****p < 0.0001. Two‐tailed unpaired Student's *t* test.

Given that DSCG treatment ameliorated depressive‐like behaviors in both LPS‐ and CRS‐induced mouse models, we subsequently examined the expression of mast cell‐associated genes in the brain. Our results displayed that the mRNA levels of Fcer1g, Fcer1a, and Fcgr4 were all upregulated in the brains of LPS‐induced depression model mice, which were all down‐regulated by DSCG administration (Figure 4A–C). Similarly, the mast cell‐specific proteases (CMA1, CPA3, and TPSB2) that were increased in depression model mice were down‐regulated by DSCG treatment as well (Figure 4D–F). Besides, DSCG administration could reduce the number of CD117^+^ cells in the brain of depression model mice (Figure 4G,H). These results collectively indicate that mast cell infiltration and activation participate in neuroinflammatory processes in LPS‐induced depression‐like mice, suggesting that DSCG may exert its antidepressant effects through modulation of the mast cell‐dependent pathway.

### 3.5 Administration of DSCG Could Reduce the Numbers of Astrocytes and Microglia in the Brains of Depression Model Mice

**FIGURE 5:**
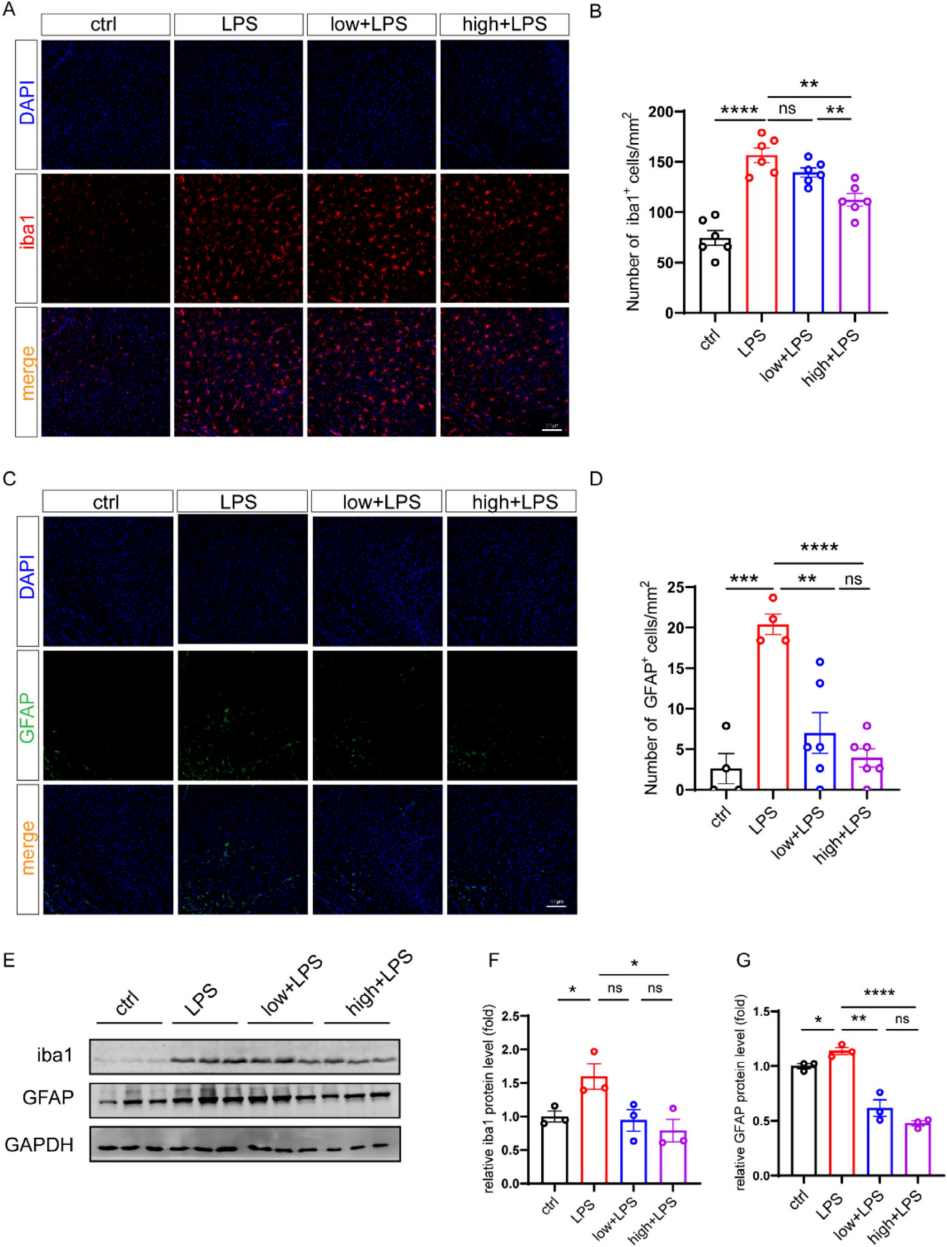
Administration of DSCG could reduce the numbers of astrocytes and microglia in the brains of depression model mice. (A) Representative immunofluorescence staining for Iba1 in the cortex. Scale bars, 100 μm. (B) Quantification of Iba1^+^ cells, including ctrl (*n* = 6), LPS (*n* = 6), low + LPS (*n* = 6), and high + LPS (*n* = 6). (C) Representative immunofluorescence staining for GFAP in the cortex. Scale bars, 100 μm. (D) Quantification of GFAP^+^ cells, ctrl (*n* = 4), LPS (*n* = 4), low + LPS (*n* = 6), and high + LPS (*n* = 6). (E) Protein levels of iba1 and GFAP in the cortex were examined by western blotting (*n* = 3 mice per group). (F, G) Normalized protein levels of iba1 and GFAP were determined by western blotting. Data are mean ± SEM. **p < 0.01, ***p < 0.001 and ****p < 0.0001. Two‐tailed unpaired Student's *t* test.

Emerging evidence indicates that both astrocytes and microglia play significant roles in the pathogenesis of depression, and that mast cell degranulation can activate these glial populations [36, 37]. To determine whether DSCG treatment affects glial activation, we quantified the number of activated astrocytes and microglia in depression model mice treated with vehicle or DSCG. Firstly, the results indicated that the number of Iba1^+^ (microglia) cells was markedly increased in the DG, CA1, and cortex of depression model mice, which were mitigated by administration of DSCG (Figure 5A,B, Figure S1). Secondly, the increasing number of GFAP^+^ (astrocyte) cells in the DG, CA1, and cortex of depression model mice was also ameliorated by administration of DSCG (Figure 5C,D, Figure S2). Last, the results of western blot further confirmed that administration of DSCG could attenuate LPS‐induced upregulation of iba1 and GFAP in the brain from depression model mice (Figure 5E–G). Collectively, these findings suggest that administration of DSCG could reduce glial activation, thereby alleviating neuroinflammatory responses in depression model mice.

### 3.6 Administration of DSCG Significantly Reduced the Expression of Pro‐Inflammatory Factor in the Brain of Depression Model Mice

**FIGURE 6:**
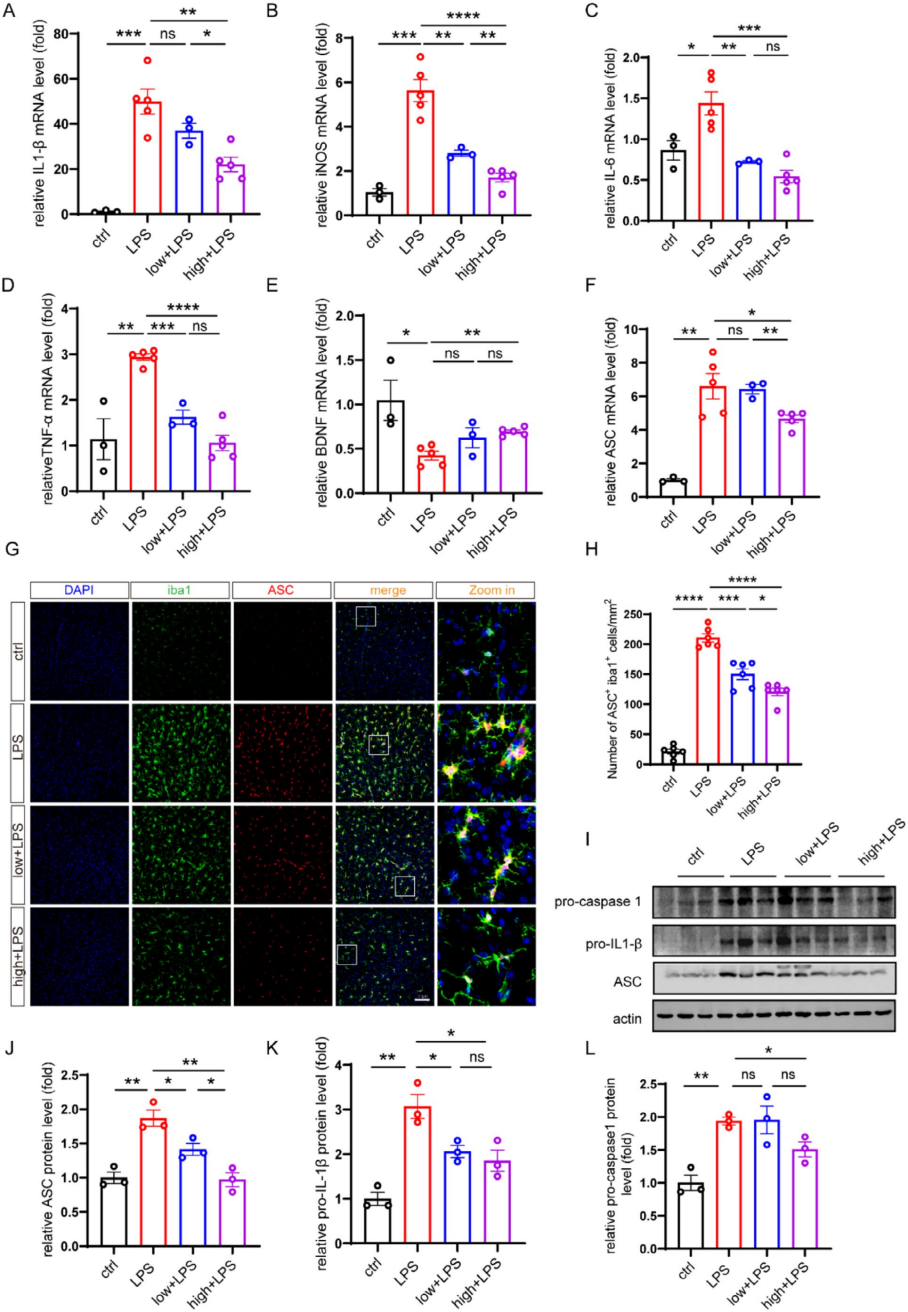
Administration of DSCG significantly reduced the expression of pro‐inflammatory factors in the brain of depression model mice. (A–F) The mRNA levels of IL‐1β, iNOS, IL‐6, TNF‐α, BDNF, and ASC in the cortex from ctrl (*n* = 3), LPS (*n* = 5), 1 mg/kg DSCG + LPS (low + LPS) (*n* = 3), and 2 mg/kg + LPS (high + LPS) (*n* = 5) mice were determined by qPCR. (G) Representative immunofluorescence staining for Iba1 and ASC in the cortex. Scale bars, 100 μm. (H) Quantification of Iba1^+^ASC^+^ cells (*n* = 6 mice per group). (I) Protein levels of pro‐caspase‐1, pro‐IL‐1β, and ASC in the cortex were examined by western blotting (*n* = 3 mice per group). (J–L) Normalized protein levels of pro‐caspase‐1, pro‐IL‐1β, and ASC were determined by western blotting. Data are mean ± SEM. *p < 0.05, **p < 0.01, ***p < 0.001, and ****p < 0.0001. Two‐tailed unpaired Student's *t* test.

The above results indicated that administration of DSCG could mitigate the increase of glial cells in the brain of depression model mice; we then want to ask whether administration of DSCG could inhibit the expression of pro‐inflammatory factors in the brain of depression model mice. The results showed that the mRNA levels of IL‐1β, iNOS, IL‐6, and TNF‐α were increased in the brain of depression model mice, and all of them were down‐regulated in the DSCG‐treated depression model mice (Figure 6A–D). Considering the well‐documented involvement of neurotrophic factors in neuronal plasticity and depression pathophysiology, we next quantified brain‐derived neurotrophic factor (BDNF) expression. Consistent with clinical observations showing decreased BDNF protein levels in the hippocampus and medial prefrontal cortex of patients with major depressive disorder [38], our experiments revealed that LPS treatment significantly suppressed BDNF expression. Notably, DSCG (2 mg/kg) effectively reversed this suppression, restoring BDNF to near‐normal levels (Figure 6E). To corroborate these transcriptional findings, we measured cytokine protein levels in brain homogenates using ELISA. Consistent with the change in mRNA levels, the ELISA measurements confirmed that DSCG administration significantly attenuated LPS‐induced elevations in the protein levels of IL‐6, TNF‐α, and IL‐1β in brain homogenates (Figure S3A–C). Taken together, these findings suggest that DSCG could reduce pro‐inflammatory factor expression in depression model mice.

### 3.7 Administration of DSCG Could Attenuate the Activation of NLRP3 Inflammasome in the Brain From Depression Model Mice

Our previous study reveals that the NLRP3 inflammasome‐mediated neuroinflammation is involved in the development of depression [39], in which the adaptor protein ASC acts as a critical linker within the inflammasome complex. We then determined the expression of ASC. The results revealed LPS significantly elevated the mRNA levels of ASC in mouse brain, an effect that was substantially reversed by DSCG treatment at 2 mg/kg (Figure 6F). Consistently, LPS‐induced increasing of the number of Iba1^+^ASC^+^ microglia in brain was also mitigated by DSCG treatment (Figure 6G,H, Figure S3D–G). The results of western blot confirmed that administration of DSCG could attenuate LPS‐induced upregulation of ASC, pro‐caspase‐1, and pro‐IL‐1β in the brain from depression model mice (Figure 6I–L). Collectively, these results indicate that DSCG suppresses inflammasome activation by downregulating ASC expression and associated signaling components in the brains of depression model mice.

## 4 Discussion

Emerging evidence implicates neuroinflammation plays a critical role in the pathogenesis of depression. MCs, as secretory immune cells, actively participate in inflammatory processes and are thought to potentiate glial cell activation [40, 41]. DSCG, a well‐characterized mast cell stabilizer, could inhibit mast cell degranulation and thereby suppress the release of pro‐inflammatory mediators [42]. In the current study, our results show that the mast cell‐associated genes were upregulated in the brain of depression model mice. Administration of DSCG could ameliorate the depressive‐like behaviors in both LPS‐ and CRS‐induced depression model mice. Mechanically, DSCG down‐regulated MCs‐associated gene expression and attenuated neuroinflammatory responses, suggesting that its antidepressant‐like effects are mediated through mast cell stabilization.

Upon degranulation, MCs rapidly release preformed inflammatory mediators, which are known to participate in the selective recruitment, activation, and regulation of immune cells (e.g., neutrophils, eosinophils, dendritic cells, and T lymphocytes) [43]. For example, the activated mast cells could release leukotriene B4 to entrap neutrophils within them, forming “Mast Cell intracellular Traps (MITs)” and leading them to death [44]. Moreover, several lines of evidence suggest that mast cells can greatly modify B cell generation and activities, produce substantial amounts of cytokines, such as IL‐6, with profound impacts on B cell development, class‐switch recombination events, and subsequent antibody production [45]. Besides, MCs‐derived products, such as CCL5 and TNF‐α, modulate the migration and function of CD8 T cells. Conversely, activated CD8 T cells induce the upregulation of MCs costimulatory molecules [46]. These results indicate that MCs could play vital roles as effector and/or immunoregulatory cells in the immune system. Furthermore, mast cells are suggested to be a key regulator of neuroinflammation [47], which serve as the brain's first responders among immune‐related cells and can trigger inflammatory cascades via degranulation [48]. Notably, emerging studies suggest bidirectional communication between mast cells and microglia [49, 50], wherein mast cell‐derived mediators such as histamine [51] and trypsin [52, 53, 54] promote microglial activation, thereby exacerbating neuroinflammation. In our study, we observed that the amount of mast cell was increased in the brain from LPS‐induced depression model mice. Administration of DSCG could down‐regulate MCs‐associated genes in the brain of depression model mice, suggesting that its antidepressant properties may stem from the suppression of mast cell‐mediated neuroinflammatory responses. Specifically, LPS‐induced mice exhibited pronounced microglia and astrocyte activation, as evidenced by elevated Iba1 and GFAP expression in CA1, DG, and cortex. Treatment with DSCG mitigated neuroinflammation by inhibiting MCs degranulation and reducing both glial activation and proliferation, supporting the notion that mast cell inhibition dampens neuroimmune activation in depression. Thus, DSCG may modulate critical neuroimmune interactions, which could underlie the broader anti‐inflammatory and psychotropic effects observed in our study.

DSCG is a classical MCs stabilizer known to prevent antigen‐ or compound 48/80‐induced degranulation by forming a ternary complex with calcium‐binding proteins on the mast cell membrane and calcium ions, thereby stabilizing the membrane and blocking exocytosis [55]. More recently, DSCG has been reported to act as an agonist at GPR35, a G protein–coupled receptor expressed in human mast cells and upregulated following IgE sensitization [56, 57]. Importantly, the anti‐allergic effects of DSCG are abolished in GPR35^−^/^−^ mice, suggesting that GPR35 is a likely molecular target of DSCG [58]. Thus, it is highly plausible that DSCG does not act on a single target but rather exerts its mast cell‐stabilizing effects through the synergistic interplay of multiple mechanisms. Future biochemical and genetic studies are warranted to elucidate the synergistic interactions among these multiple targets of DSCG within the brain.

The NLRP3 inflammasome is a multiprotein complex that initiates inflammatory cytokine production upon activation, plays a critical role in the development of major depression [59]. Within this complex, the adaptor protein ASC serves as a critical linker, bridging NLRP3 with pro‐caspase‐1 to form the functional NLRP3‐ASC‐pro‐caspase‐1 inflammasome. Our results demonstrated DSCG treatment markedly suppressed the expression of ASC in the brain of depression model mice, which further indicates that administration of DSCG could attenuate the neuroinflammation in the brain of depression model mice. Although DSCG treatment reduced ASC in our model, consistent with prior studies [50], there is currently no direct evidence supporting that DSCG interacts with NLRP3 or ASC.

While the clinical use of DSCG in classical indications such as asthma has declined with the advent of targeted biologics, its favorable safety profile and low systemic toxicity make it an attractive pharmacological tool for exploring mast cell– and neuroimmune‐mediated mechanisms. Beyond its traditional anti‐allergic roles, emerging evidence indicates that DSCG may exert neuroprotective and anti‐inflammatory effects in conditions such as chronic pruritus and neuroinflammatory disorders [60]. These findings highlight the potential for systematic re‐evaluation of DSCG's molecular targets, which may facilitate drug repurposing toward novel therapeutic indications, including depression.

In summary, our study demonstrates that DSCG suppresses neuroinflammation and alleviates depressive‐like behaviors primarily through mast cell stabilization and downregulation of glial activation. Elucidating the precise molecular interactions underlying these effects will be critical for rationally repositioning DSCG and for developing next‐generation selective modulators of MCs–microglia–neuroinflammation pathways as potential therapeutics for inflammation‐associated depression.

## Author Contributions

Y.L. and Z.Y. designed the experiments, Y.X. and K.L. performed the experiments and analyzed the data, Y.X. wrote the manuscript. Y.L. and Z.Y. revised the manuscript. All authors have reviewed the manuscript.

## Funding

This work was supported by grants from the National Natural Science Foundation of China (82271236 to Y.J.L., and 82230042 to Z.Y.), the Science and Technology Innovation Program of Hunan Province (2022RC1219 to Y.J.L.), Hunan Provincial Natural Science Foundation of China (2025JJ50536 to Y.J.L.), University of South China Clinical Research 4310 Program (20224310NHYCG08 to Y.J.L.), and the Clinical Medical Research Center of Hunan Province (2023SK4050 to Y.J.L.).

## Ethics Statement

All experimental animal procedures were approved by the Institutional Animal Care and Use Committee of the Beijing Institute of Basic Medical Sciences (Approval Number: AF/SC‐08/02.320).

## Conflicts of Interest

The authors declare no conflicts of interest.

## Supporting information


**Table S1:** The primer sequences for qPCR.
**Figure S1:** Administration of DSCG could decrease the number of microglia in the brain of depression mice. (A, C) Representative immunofluorescence staining for Iba1 in the DG and CA1. Scale bars, 100 μm. (B, D) Quantification of Iba1^+^ cells in the DG (B) and CA1 (D) (*n* = 6 mice per group). Data are mean ± SEM. *p < 0.05, **p < 0.01, ***p < 0.001 and ****p < 0.0001. Two‐tailed unpaired Student's *t* test.
**Figure S2:** Administration of DSCG could decrease the number of astrocytes in the brain of depression mice. (A, C) Representative immunofluorescence staining for GFAP in the DG and CA1. Scale bars, 100 μm. (B, D) Quantification of GFAP^+^ cells in the DG (B) and CA1 (D), ctlr (*n* = 4), LPS (*n* = 4), low + LPS (*n* = 6), and high + LPS (*n* = 6). Data are mean ± SEM. *p < 0.05, **p < 0.01. Two‐tailed unpaired Student's *t* test.
**Figure S3:** Administration of DSCG could suppress the expression of pro‐inflammatory factors in LPS‐induced mice. (A–C) The concentration of IL‐6, TNF‐α and IL‐1β in the brain homogenate (*n* = 6 mice per group), measured by ELISA. (D, F) Representative immunofluorescence staining for Iba1 and ASC in the DG (D) and CA1 (F). Scale bars, 100 μm. (E, G) Quantification of Iba‐1^+^ASC^+^ cells in the DG (E) and CA1 (G) (*n* = 6 mice per group). Data are mean ± SEM. *p < 0.05, **p < 0.01, ***p < 0.001 and ****p < 0.0001. Two‐tailed unpaired Student's *t* test.

## Data Availability

The data that support the findings of this study are available from the corresponding author upon reasonable request.
