# Supplementary material for: Disodium Cromoglycate Attenuates the Depressive‐Like Behaviors in Mice by Inhibiting Neuroinflammation
Source: CNS Neurosci Ther. 2026 Jan 20;32(1):e70721. doi: 10.1002/cns.70721 (PMC12817297; doi:10.1002/cns.70721)
Supplement: Supplementary file 1 — Table S1: The primer sequences for qPCR. Figure S1: Administration of DSCG could decrease the number of microglia in the brain of depression mice. (A, C) Representative immunofluorescence staining for Iba1 in the DG and CA1. Scale bars, 100 μm. (B, D) Quantification of Iba1+ cells in the DG (B) and CA1 (D) (n = 6 mice per group). Data are mean ± SEM. *p < 0.05, **p < 0.01, ***p < 0.001 and ****p < 0.0001. Two‐tailed unpaired Student's t test. Figure S2: Administration of DSCG could decrease the number of astrocytes in the brain of depression mice. (A, C) Representative immunofluorescence staining for GFAP in the DG and CA1. Scale bars, 100 μm. (B, D) Quantification of GFAP+ cells in the DG (B) and CA1 (D), ctlr (n = 4), LPS (n = 4), low + LPS (n = 6), and high + LPS (n = 6). Data are mean ± SEM. *p < 0.05, **p < 0.01. Two‐tailed unpaired Student's t test. Figure S3: Administration of DSCG could suppress the expression of pro‐inflammatory factors in LPS‐induced mice. (A–C) The concentration of IL‐6, TNF‐α and IL‐1β in the brain homogenate (n = 6 mice per group), measured by ELISA. (D, F) Representative immunofluorescence staining for Iba1 and ASC in the DG (D) and CA1 (F). Scale bars, 100 μm. (E, G) Quantification of Iba‐1+ASC+ cells in the DG (E) and CA1 (G) (n = 6 mice per group). Data are mean ± SEM. *p < 0.05, **p < 0.01, ***p < 0.001 and ****p < 0.0001. Two‐tailed unpaired Student's t test. [file CNS-32-e70721-s001.docx]

**Table S1: The primer sequences for qPCR**

| Gene | Forward | Reverse |
| --- | --- | --- |
| β-actin | CATTGCTGACAGGATGCAGAAGG | TGCTGGAAGGTGGACAGTGAGG |
| IL-6 | TACCACTTCACAAGTCGGAGGC | CTGCAAGTGCATCATCGTTGTTC |
| iNOS | GAGACAGGGAAGTCTGAAGCAC | CCAGCAGTAGTTGCTCCTCTTC |
| TNF-a | GGTGCCTATGTCTCAGCCTCTT | GCCATAGAACTGATGAGAGGGAG |
| IL-1β | TGGACCTTCCAGGATGAGGACA | GTTCATCTCGGAGCCTGTAGTG |
| ASC | CTGCTCAGAGTACAGCCAGAAC | CTGTCCTTCAGTCAGCACACTG |
| BDNF | GGCTGACACTTTTGAGCACGTC | CTCCAAAGGCACTTGACTGCTG |
| Fcer1a | GTCTCCATTAGAGAGGCCACAC | AGAGCAATAACCCCGTGTCC |
| Fcer1g | ATCTCAGCCGTGATCTTGTTCT | ACCATACAAAAACAGGACAGCAT |
| Fcgr4 | ATGTGGCAGCTACTACTACCA | ACCCACTTGGGGTCTAGGTTC |
| TPSB2 | GGAGGTTCTCTCATCCATCCAC | CCTGTTCAAAGAGAGGAGCTGG |
| CPA3 | CAAACTGCCTCCTAACCACCAG | CCAGTCTAAGGAAGAGCCTGAAG |
| CMA1 | TCTCCTGGGTTCCAGCACCAAA | GCCGACAGGTAGTTCTCAGAAG |


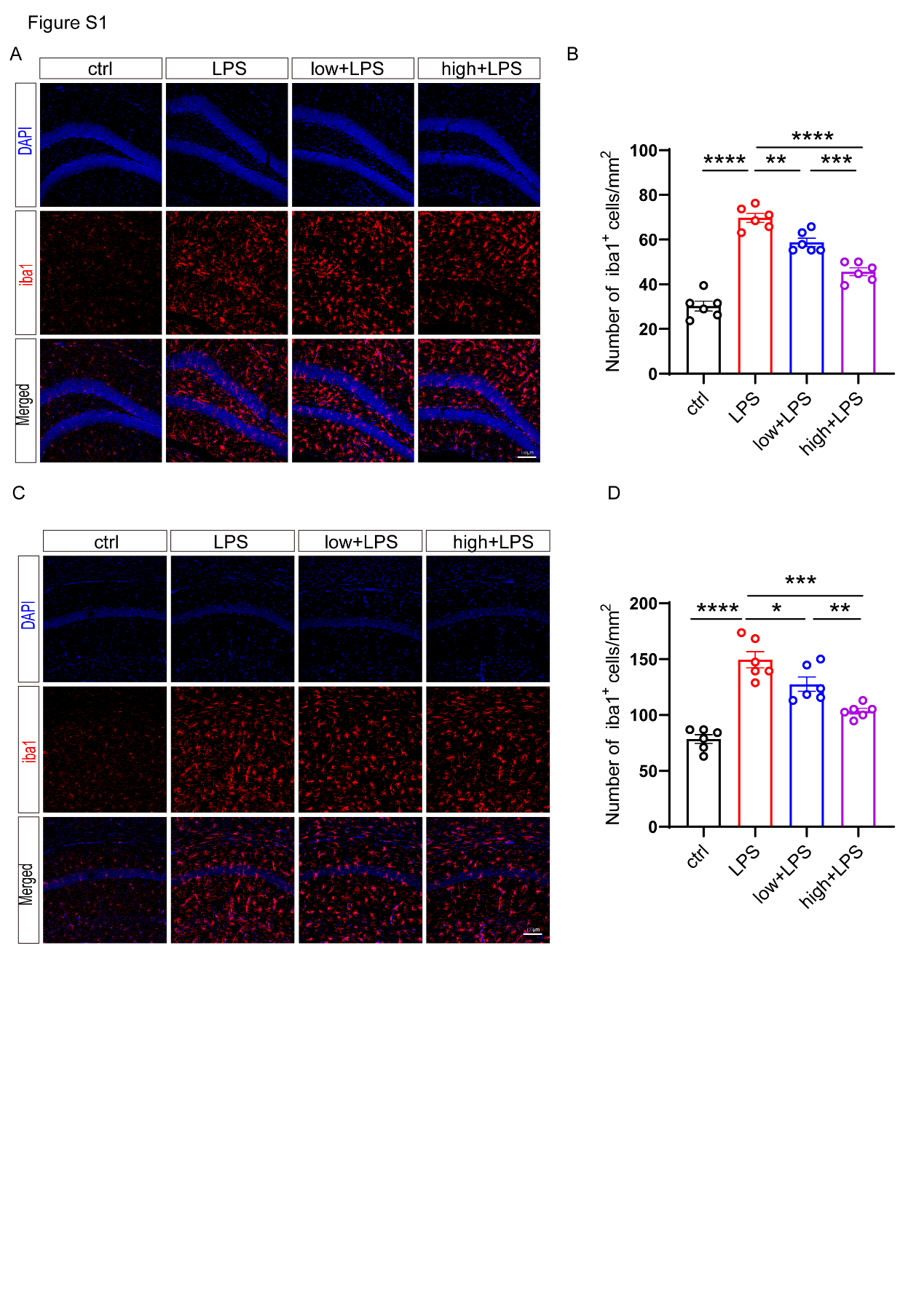


**Figure S1: Administration of DSCG could decrease the number of microglia in the brain of depression mice.** (A, C) Representative immunofluorescence staining for Iba1 in the DG and CA1. Scale bars, 100 μm. (B, D) Quantification of Iba1^+^ cells in the DG (B) and CA1 (D) (n = 6 mice per group). Data are mean ± SEM. *p < 0.05, **p < 0.01, ***p < 0.001 and ****p < 0.0001. Two-tailed unpaired Student’s t test.


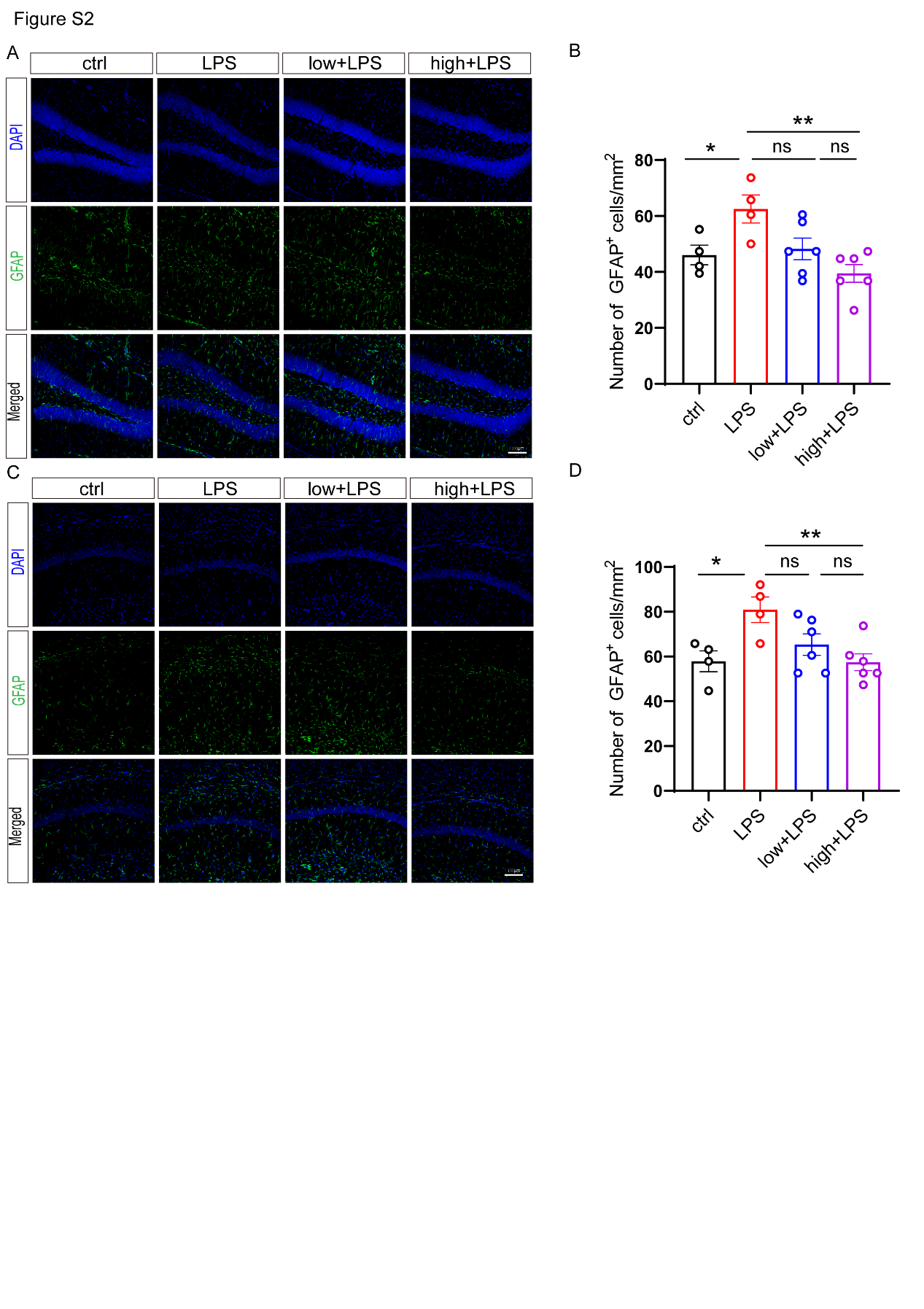


**Figure S2: Administration of DSCG could decrease the number of astrocytes in the brain of depression mice.** (A, C) Representative immunofluorescence staining for GFAP in the DG and CA1. Scale bars, 100 μm. (B, D) Quantification of GFAP^+^ cells in the DG (B) and CA1 (D), ctlr (n = 4), LPS (n = 4), low+LPS (n = 6), and high+LPS (n = 6). Data are mean ± SEM. *p < 0.05, **p < 0.01. Two-tailed unpaired Student’s t test.


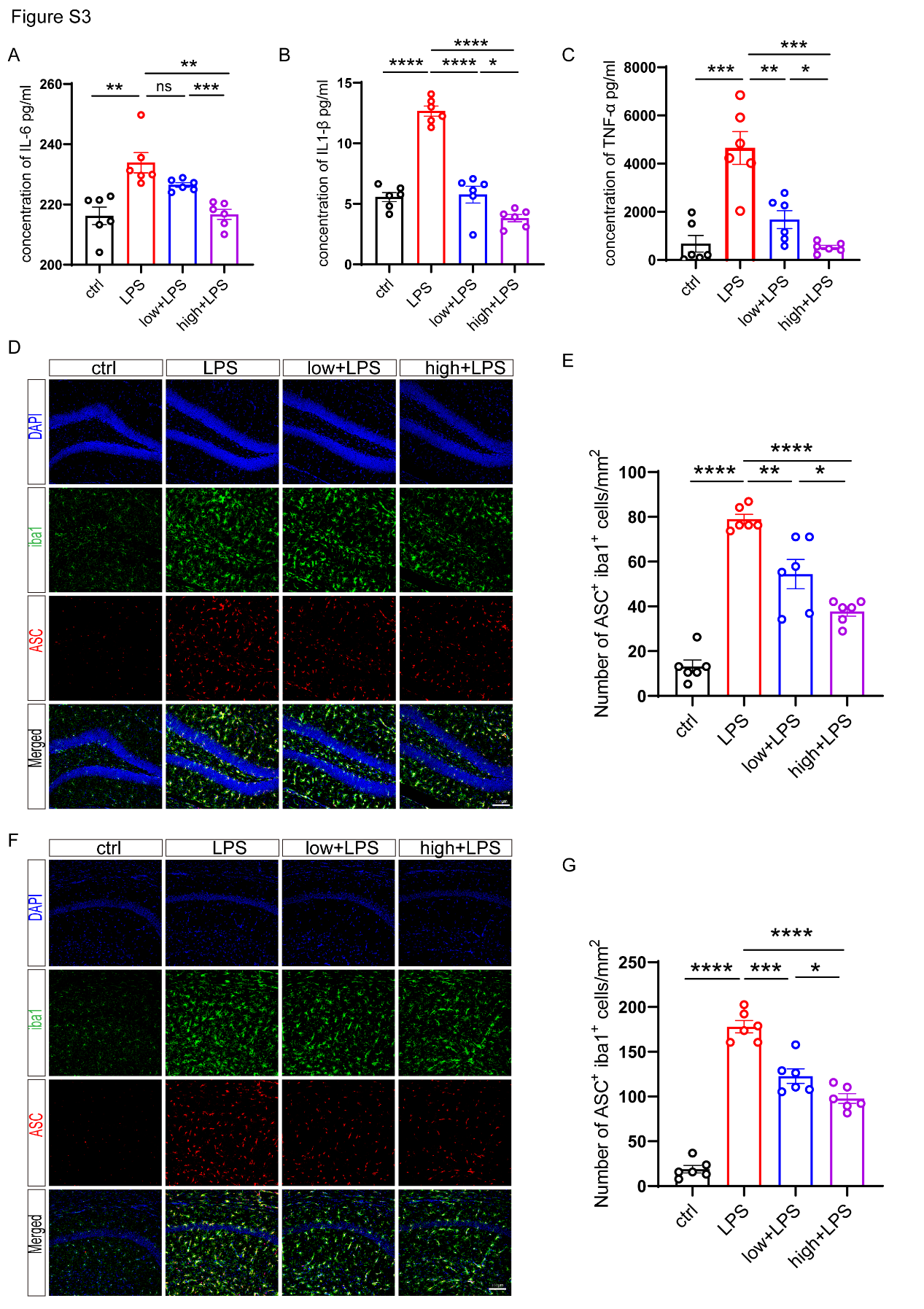


**Figure S3: Administration of DSCG could suppress the expression of pro-inflammatory factors in LPS-induced mice.** (A-C) The concentration of IL-6, TNF-α and IL-1β in the brain homogenate (n = 6 mice per group), measured by ELISA. (D, F) Representative immunofluorescence staining for Iba1 and ASC in the DG (D) and CA1 (F). Scale bars, 100 μm. (E, G) Quantification of Iba-1^+^ASC^+^ cells in the DG (E) and CA1 (G) (n = 6 mice per group). Data are mean ± SEM. *p < 0.05, **p < 0.01, ***p < 0.001 and ****p < 0.0001. Two-tailed unpaired Student’s t test.
